# Mildly Increased Renin Expression in the Absence of Kidney Injury in the Murine Transverse Aortic Constriction Model

**DOI:** 10.3389/fphar.2021.614656

**Published:** 2021-06-15

**Authors:** Marian Wesseling, Eva Mulder, Maike A. D. Brans, Daniek. M. C. Kapteijn, Marian Bulthuis, Gerard Pasterkamp, Marianne C. Verhaar, A. H. Jan Danser, Harry van Goor, Jaap A. Joles, Saskia C. A. de Jager

**Affiliations:** ^1^ Laboratory for Experimental Cardiology, University Medical Center Utrecht, Utrecht, Netherlands; ^2^ Laboratory for Clinical Chemistry and Haematology, University Medical Center Utrecht, Utrecht, Netherlands; ^3^ Pathology and Medical Biology, University Medical Center Groningen, Groningen, Netherlands; ^4^ Nephrology and Hypertension, University Medical Center Utrecht, Utrecht, Netherlands; ^5^ Department of Pharmacology, Erasmus University Medical Center Rotterdam, Rotterdam, Netherlands; ^6^ Laboratory for Translational Immunology, University Medical Center Utrecht, Utrecht, Netherlands

**Keywords:** transverse aortic constriction, kidney function, cardiorenal syndrome, stroke volume, hypoperfusion, murine (mouse), renin expression

## Abstract

Cardiorenal syndrome type 2 is characterized by kidney failure as a consequence of heart failure that affects >50% of heart failure patients. Murine transverse aortic constriction (TAC) is a heart failure model, where pressure overload is induced on the heart without any systemic hypertension or its consequences. Whether renal function is altered in this model is debated, and if so, at which time post-TAC renal dysfunction starts to contribute to worsening of cardiac function. We therefore studied the effects of progressive heart failure development on kidney function in the absence of chronically elevated systemic blood pressure and renal perfusion pressure. C57BL/6J mice (N = 129) were exposed to TAC using a minimally invasive technique and followed from 3 to 70 days post-TAC. Cardiac function was determined with 3D ultrasound and showed a gradual decrease in stroke volume over time. Renal renin expression and plasma renin concentration increased with progressive heart failure, suggesting hypoperfusion of the kidney. In addition, plasma urea concentration, a surrogate marker for renal dysfunction, was increased post-TAC. However, no structural abnormalities in the kidney, nor albuminuria were present at any time-point post-TAC. Progressive heart failure is associated with increased renin expression, but only mildly affected renal function without inducing structural injury. In combination, these data suggest that heart failure alone does not contribute to kidney dysfunction in mice.

## Introduction

Cardiorenal syndrome (CRS) is a clinical characterization of patients with both heart and kidney failure where CRS type 2 describes chronic kidney disease (CKD) as consequence of heart failure (Zannad and Rossignol, 2018; Rangaswami et al., 2019). Especially in the non-ischemic heart failure population, patients often suffer from CKD (van Deursen et al., 2014; Schefold et al., 2016). Indeed, over 50% of all heart failure patients show a certain degree of CKD which has been associated with increased mortality rates (Hillege et al., 2006; van Deursen et al., 2014).

A generally accepted model to study adverse cardiac remodeling in heart failure is transverse aortic constriction (TAC). Cardiac remodeling in this model is characterized by early structural changes due to inflammation and fibrosis, and results in left ventricular hypertrophy (Furihata et al., 2016; Gross, 2019). Consequently, the functional capacity of the heart deteriorates as end systolic and diastolic volumes increase and stroke volume is reduced (Hampton et al., 2017). As the kidneys are downstream of the heart, it is reasonable to assume that they experience hypoperfusion in the TAC model, due to a reduced stroke volume. From the two kidney one clip model (2K1C) it has been established that renal hypoperfusion leads to local renin-angiotensin system (RAS) stimulation, consequently leading to various systemic effects, including aldosterone release (Krebs et al., 2007). Little information is available on the progressive deterioration of kidney function in the TAC model, although RAS stimulation has been documented to exacerbate heart failure progression after TAC (Rockman et al., 1994; Li et al., 2018).

TAC has been proven to be a relevant preclinical model for chronic non-ischemic heart failure; however, for CRS Type 2 this is debated (Kamal et al., 2017; Li et al., 2018; Tan et al., 2018; Richards et al., 2019; Zhao et al., 2019). Based on the reduced stroke volume in the TAC model and increased renin expression in the 2K1C model, we hypothesized that in the TAC model, hypoperfusion in the kidneys directly leads to renin activation and in the long-term renal hypofiltration, fibrosis and atrophy, as has been shown by us and others for the clipped kidney in the 2K1C model (Krebs et al., 2007; Cheng et al., 2009). To identify acute and progressive effects of heart failure on the kidney, we aimed to establish the structural and functional deterioration of the kidney in a mouse TAC model. Since systemic hypertension, and thus increased renal perfusion pressure, is absent in the TAC model, it enables us to distinguish the direct effects of acute and chronic heart failure on acute and chronic kidney injury (Han and Ryu, 2011; ter Maaten et al., 2016).

## Materials and Methods

### Animals

Male and female C57BL/6J mice were used, originally obtained from the Jackson laboratory and kept in our breeding facility. Animals were conventionally housed, separated on sex, in groups of maximum six animals at room temperature in type III filter top cages with Aspen Woodchip bedding, a plastic shelter with a light/dark cycle of 12/12 h under strict DM1 regulations and received standard chow and water ad libitum. Mice were visually monitored daily and cages were cleaned once weekly. Researchers and technicians were blinded for animal groups, experimental procedures, data acquisition and analysis. Mice, aged 9–12 weeks, were included in the study with a starting weight between 20 and 30 g. All mice were randomly assigned to follow-up time after TAC as described (De Haan et al., 2017). To determine group size, we used kidney function based on increased urea levels as primary outcome measure. For the power calculation we used G*power software (version 3.1.9, Heinrich Heine University Dusseldorf, Germany), we calculated a minimum of five animals per sex and per timepoint. Given the progressive development of cardiac dysfunction over time in this model combined with the number of timepoints (9 timepoints during follow-up) included in this study, this should allow us to dissect changes in kidney function. These changes will be assessed up to an endpoint of 70 days, this specific end point correlates to the timeline manifestations of CRS2 previously observed in TAC mice (Kamal et al., 2017; Zhao et al., 2019). Furthermore, beyond 70 days TAC we risk the loss of a substantial number of experimental animals due to the development of severe heart failure. An experimental group without TAC terminated at day 0 of the study was included as control group. Supplementary Figure S1A shows a flowchart with animals (experimental unit) used per experimental group, mice excluded based on flow ratios (*n* = 10), and mice lost during follow up (*n* = 12). Mortality only occurred within 7 days post-TAC and was most likely a direct consequence of the TAC surgery and not of the development of heart failure. We included a total of 129 mice and finally analyzed 13 baseline (no surgery) and 94 TAC mice.

### Transverse Aortic Constriction

All animal experiments were performed according to the “Guide for the care and use of Laboratory Animals”. Experiments were approved by the Animal Experiments Committee of the University Medical Center Utrecht (Utrecht, Netherlands) and reported according to the Arrive guidelines (Sert et al., 2019). Surgery was performed by an experienced surgeon in a dedicated mouse operation room. Mice were anesthetized by intraperitoneal (i.p.) injection of medetomidine hydrochloride (SEDAStart, 1.0 g/kg body weight, AST Farma, Netherlands), midazolam (1.0 mg/kg body weight, Actavis Group PTC, Iceland) and fentanyl (0.1 mg/kg, Bipharma, Netherlands). Mice were intubated and ventilated on a rodent ventilator (Minivent, Hugo Sachs Electronics, Germany) with an oxygen-air ratio of 1:1 (175 strokes/minute, 250 μl stroke volume). *Via* a thoracic incision between the upper left sternal border in the second intercostal space, a ligature was placed around the transverse aorta between the right and left common carotid arteries. Constriction was standardized by placing a 7–0 silk suture around a blunt 27-gauge needle which was subsequently removed. Surgery time is kept as short as possible and a subcutaneous injection of anesthetic antagonist Atipam (1.0 mg/kg, Dechra Veterinary Products, Netherlands), Flumazenil (0.5 mg/kg, B. Braun, Germany) and pain killer Buprenorphine (0.1 mg/kg, Temgesic, Indivior United Kingdom) is directly provided upon finalizing the surgical procedure. Post-surgery the animals return to their cages and were kept on a heating pad for 24 h. Mice received Buprenorphine up till two days post-surgery with a 12 h time interval for optimal pain relieve.

### Echocardiography

For transthoracic 3D-echocardiography, we used the Vevo 2100 System with a 22–35 MHz transducer (MS550D; VisualSonics Inc., Toronto, Canada), to assess cardiac function by structural and functional parameters. The mice underwent echocardiography at baseline, day 7 and at termination, under the inhalation of 2% isoflurane in a mixture of oxygen/air of 1:1. Induction of anesthesia is conducted with an induction chamber to minimize stress and discomfort. At day 7 echocardiography was performed to confirm correct placement of the ligation by Doppler flow measurements on the carotid arteries (Supplementary Figure S1B). Only animals with a flow ratio (between left and right carotid) >5 were included in the study. At baseline and termination, two-dimensional echocardiography images were recorded on the short and long axis of the heart at multiple levels in both end systole and end diastole with use of respiratory triggering. The VevoLab software (Fujifilm; VisualSonics Inc.) was used for analyses on cardiac flow and volumes.

### Tissue Collection

Mice were sacrificed at baseline, 3, 7, 14, 21, 28, 35, 42, 56, or 70 days after TAC (Supplementary Figure S1C). When possible, urine was collected directly before termination, by fixation of the mice and bladder stimulation by hand, and stored at −20^o^C until further analysis. Mice were terminated by exsanguination after i.p. administration of overdose sodium pentobarbital (60 mg/kg, Veterinary Medicine Pharmacy, Utrecht, Netherlands). Blood was collected in EDTA-coated tubes *via* orbital puncture for plasma collection and stored at −80^o^C until further analysis. The vascular system was flushed with 5 ml phosphate-buffered saline (PBS) *via* right ventricular puncture. Heart weight and tibia length was assessed. The kidneys were cut in half longitudinally, and half of the kidney was snap-frozen in liquid nitrogen for RNA isolation. The other half was fixed in 4% paraformaldehyde for 24 h and subsequently embedded in paraffin for histology.

### Urea and Renin Measurements in Plasma

In the plasma samples we determined urea levels with the Urea CT* FS** according to manufacturer’s protocol (DiaSys Diagnostic Systems, GmbH, Germany). Renin concentrations in plasma were determined by enzyme-kinetic assay, by quantifying Ang I generation in the presence of excess angiotensinogen (van Thiel et al., 2017).

### Albumin Measurements in Urine

In order to measure albumin levels in urine animals were placed in metabolic cages, allowing separate collection of urine overnight (15 h, from 17:00 to 08:00) to collect urine at day 3, 7, and 14. Before placement in metabolic cages animals underwent a TAC procedure as described above. Mice put on 15% glucose supplemented water overnight in order to increase drinking and consequential urine secretion. Mice were also deprived of food during this period. On day 14 animals were sacrificed and urine was collected and stored at −20^o^C. Albumin levels were determined using a Mouse Albumin Elisa kit according to manufacturer’s protocol (Bethyl laboratories Inc., Bioke, Leiden, Netherlands). To correct for variation in urine concentration, urine creatinine levels were measured with the Creatinine PAP FS* according to the manufacturer’s protocol (DiaSys Diagnostic Systems, GmbH, DE).

### Histology

From the paraffin embedded kidneys, 3 μm sections were cut and fixed on glass slides (X-tra^tm^ Adhesive, Surgipath, Leica Biosystems, United Kingdom). Sections are deparaffinized and stained for Periodic Acid-Schiff (PAS) to evaluate glomerular and tubular morphology. Furthermore, sections were stained for renin as described previously (Krebs et al., 2007). Both PAS and renin immunohistochemistry analysis was performed on the scanned slides with Image Scope (v12.3.2.8013 Aperio, Leica Biosystems Imaging, Inc., Buffalo Grove, IL, United States). Renin positive cells were counted in all glomeruli of one renal cross section. Subsequently, the number of renin positive cells were corrected for the total number of glomeruli per renal cross section, since the juxtaglomerular apparatus was not visible in all glomeruli. Analysis was performed in a blinded fashion by two independent observers.

### qPCR Analysis

For total RNA extraction, murine kidneys were mechanically disrupted in 1 ml TriPure (Roche), further processed to obtain RNA. 500 ng of RNA was reverse transcribed using RevertAid First Strand cDNA Synthesis Kits (Biorad, Veenendaal, Netherlands). Quantitative PCR (qPCR) experiments were performed using SYBR Green (Bio-Rad, Veenendaal, Netherlands) and a Bio-Rad CFX Connect device. Threshold cycle values (Ct) were analyzed and expression was quantified using the 2^−∆∆Ct^ method. GAPDH is used as household gene (Forward primer “gccttccgtgttcctacc” Reverse primer “gcc​tgc​ttc​acc​acc​ttc”) to correct for the expression of relative levels of Renin (Forward primer “cac​tct​tgt​tgt​ctg​gac​ct” Reverse primer “ggg​gta​cca​atg​ccg​atc​tc”), AT1 (Forward primer “aac​agc​ttg​gtg​gtg​atc​gtc” Reverse primer “cat​agc​ggt​ata​gac​agc​cca”), NGAL (Forward primer “ggg​aaa​tat​gca​cag​gta​tcc​tc” Reverse primer “cat​ggc​aac​tgg​ttg​tag​tc”), CTGF (Forward primer “gga​cac​cta​aaa​tcg​cca​agc” Reverse primer “act​tag​ccc​tgt​atg​tct​tca​ca”), ACE1 (Forward primer “cgc​acg​aca​cca​aca​tca​c” Reverse primer “gcc​aaa​tgg​act​cat​aca​act​cc”), ACE2 (Forward primer “tcc​aga​ctc​cga​tca​tca​agc” Reverse primer “gct​cat​ggt​gtt​cag​aat​tgt​gt”, AGT (Forward primer “tct​cct​tta​cca​caa​caa​gag​ca” Reverse primer “ctt​ctc​att​cac​agg​gga​ggt”) (P)RR (Forward primer “ctg​gtg​gcg​ggt​gct​tta​g” Reverse primer “gct​acg​tct​ggg​att​cga​tct”).

### Statistics

For statistical analysis, we used one-way ANOVA followed by Dunnett’s multiple comparisons test (all TAC groups were compared to the baseline group) performed with GraphPad (Prism 8.0.1 for Windows, GraphPad Software, San Diego, Canada). A one-way ANCOVA was conducted to determine a statistically significant difference between gender on cardiac and renal function over time post-TAC. Data is shown as mean ± SD. For associations we used linear regression. *p* < 0.05 was considered significant.

## Results

### Gradual Adverse Cardiac Remodeling Upon TAC

**FIGURE 1:**
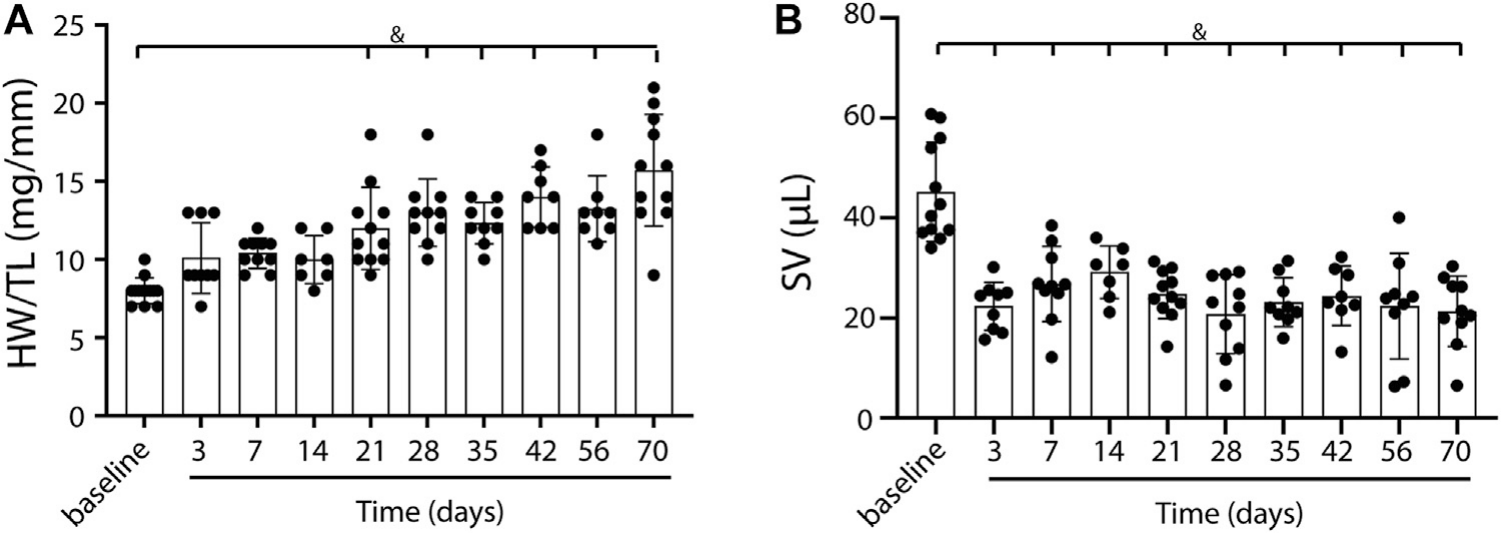
Cardiac weight and function in TAC mice over time. A gradual increase in heart weight to tibia length ratio over time, indicating the presence of cardiac remodeling post-TAC **(A)**. Stroke volume decreases over time post TAC vs. baseline **(B)**. Mean ± SD. & = *p* < 0.0001. HW/TL; Heart weight/Tibia length.

To induce heart failure, mice were subjected to pressure overload by TAC. Heart weight to tibia length ratio, determined as an indication of cardiac remodeling, was progressively and significantly increased compared to control (baseline) mice from 21 days post-TAC (*p* < 0.0001, Figure 1A). Stroke volume decreased, starting at day 3 up to day 70, but did not show a statistically significant progressive deterioration over time (Figure 1B). In line, increased end systolic volume, end diastolic volume and reduced contraction (measured as global longitudinal strain) together with a gradually decreased EF (baseline 67.99 vs. 22.17 at termination, *p* < 0.001) confirm structural remodeling with progressively reduced cardiac function over time after TAC (Table 1). We were unable to reliably calculate cardiac output in our model, as heart rate is known to be influenced by the used anesthetics and body temperature during image acquisition. Besides a deteriorating heart function, no clear signs such as cachexia (Supplementary Figure S2) or lung edema, reflected by the presence of lethargy or dyspnea, were present. We observed no sex-differences in EDV (F (1,74) = 0.241, *p* = 0.63), ESV (F (1,74) = 0.220. *p* = 0.64), EF (F (1,14) = 0.085, *p* = 0.77), and SV(F (1,92) = 0.013, *p* = 0.91).

**TABLE 1:** Echocardiography variables.

		ESV		EDV		SV		EF		Strain	
Time (days)	*n*	mean (sd)	adjusted *p* value	mean (sd)	adjusted *p* value	mean (sd)	adjusted *p* value	mean (sd)	adjusted *p* value	mean (sd)	adjusted *p* value
Baseline	13	16.24 (3.99)	–	48.57 (9.62)	–	45.23 (9.91)	–	67.99 (5.38)	–	−17.82 (1.99)	–
3	8	38.58 (11.66)	0.0068	60.66 (10.22)	0.3101	22.37 (4.81)	<0.0001	37.33 (10.58)	<0.0001	−12.96 (4.03)	0.0180
7	10	30.21 (7.36)	0.1500	56.59 (11.30)	0.7121	26.84 (7.51)	<0.0001	47.08 (8.33)	<0.0001	−10.928 (4.32)	<0.0001
14	7	28.87 (7.24)	0.2806	59.14 (10.20)	0.4246	29.17 (5.25)	0.0001	51.42 (7.60)	0.0025	−11.50 (1.23)	0.0002
21	11	44.60 (19.37)	0.0001	69.91 (19.01)	0.0035	24.77 (4.88)	<0.0001	37.94 (10.44)	<0.0001	−8.29 (2.92)	<0.0001
28	10	44.97 (17.18)	0.0001	65.74 (12.71)	0.0320	20.75 (7.86)	<0.0001	33.53 (17.63)	<0.0001	−10.80 (3.66)	<0.0001
35	9	40.74 (12.27)	0.0023	63.63 (13.68)	0.1087	23.16 (4.88)	<0.0001	36.87 (8.37)	<0.0001	−8.37 (3.22)	<0.0001
42	8	44.75 (18.66)	<0.0001	77.46 (16.20)	0.0011	24.45 (5.93)	<0.0001	40.37 (16.55)	<0.0001	−7.94 (5.19)	<0.0001
56	9	62.77 (25.51)	<0.0001	85.34 (17.42)	<0.0001	22.40 (10.54)	<0.0001	28.61 (15.73)	<0.0001	−7.25 (2.28)	<0.0001
70	11	78.87 (28.13)	<0.0001	98.76 (24.80)	<0.0001	21.34 (7.04)	<0.0001	22.17 (12.16)	<0.0001	−6.70 (3.46)	<0.0001

*p*-values are presented compared with baseline values. Abbreviations, ESV; end systolic volume, EDV; end diastolic volume, SV; stroke volume, EF; ejection fraction.

### Renin Expression and Its Association to Cardiac Function

**FIGURE 2:**
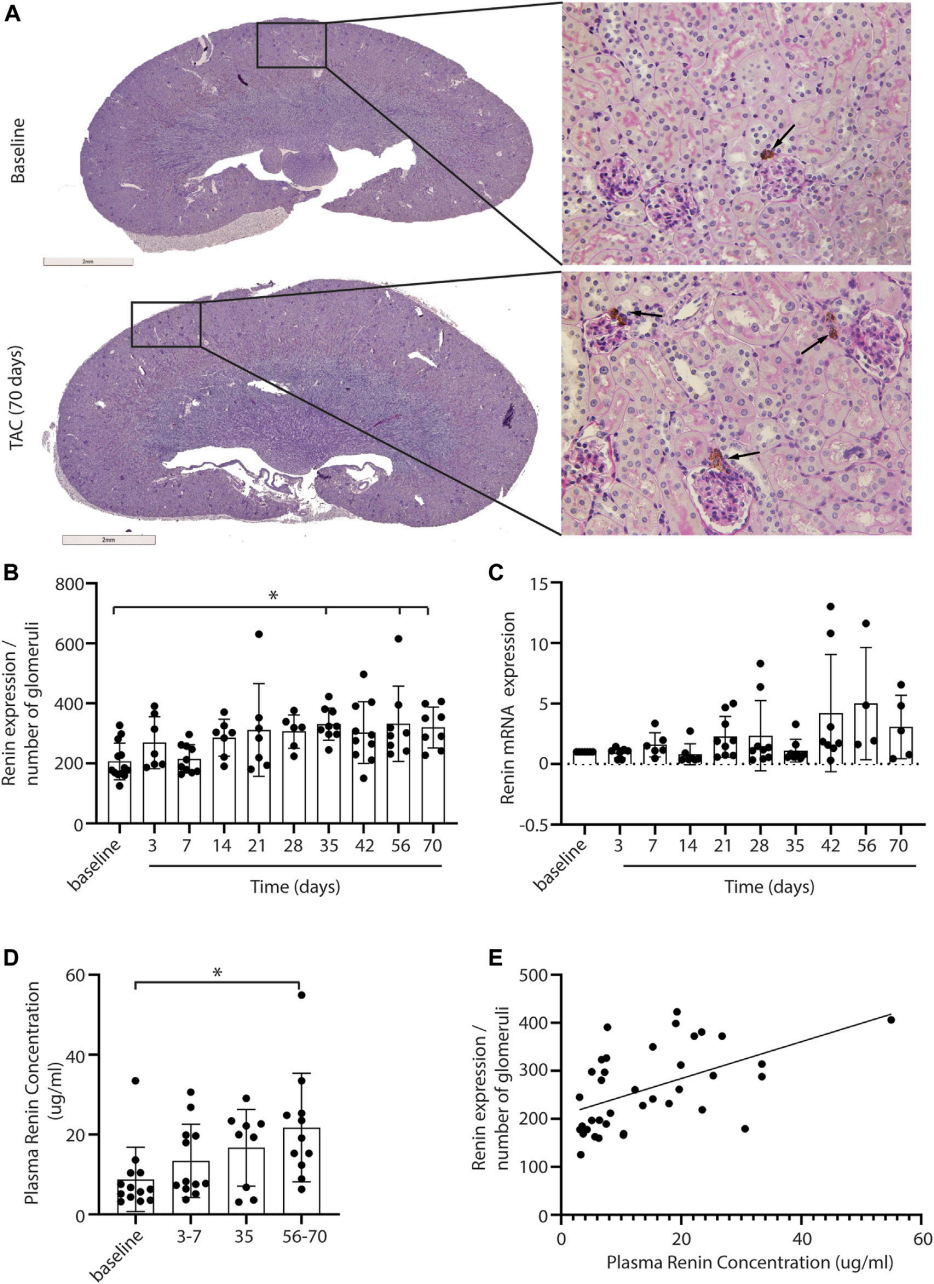
Renal renin expression after TAC. Representative pictures of renin expression at baseline and after 3–70 days of TAC **(A)**. Renin expression (depicted in brown) is visible in the juxtaglomerular cells close to the glomeruli (arrows). Renin expression, corrected for the number of glomeruli present in the section, is increased from day 35 compared to baseline **(B)**. qPCR analysis of renin expression showed a trend of increased renin over time **(C)**, 20 mice were excluded from the renin qPCR analysis as the levels were below the detection limit. In line with renal renin, the plasma renin concentration is increased significantly at 56–70 days post-TAC **(D)**. A significant association between local and systemic renin is observed (r = 0.52, R2 = 0.27, *p* = < 0.001) **(E)**. Mean ± SD, * = *p* < 0.05.

A decrease in stroke volume can lead to hypoperfusion of the kidney and consequently affect renal function. With immunohistochemistry, renin expression in renal juxtaglomerular cells was evaluated to establish RAS activation in response to the possible reduced blood flow upon TAC (Figure 2A, black arrows indicate renin staining). Analysis showed a mild but progressive increase of renin in the juxtaglomerular apparatus, reaching statistical significance from 35 days post-TAC vs. control (day 35, 56, and 70 *p* = < 0.05) (Figure 2B). With qPCR we found a trend of increased renin mRNA expression over time (Figure 2C). To see whether a local increase in renin also resulted in a systemic increase in renin (and thus RAS upregulation), we measured the plasma renin concentration and observed a significant increase after 56–70 days (*p* = < 0.01) (Figure 2D). Importantly, the systemic levels of renin correlated to the local increase in renal renin expression post-TAC (r = 0.52, R2 = 0.27, *p* = < 0.001) (Figure 2E). Taken together, these results are suggestive for gradually increased renal renin expression, leading to the release of more renin into the systemic circulation, and thus increased RAS activity. qPCR analysis of other RAS components in the kidney showed no significant differences in angiotensin two receptor type 1 (AT1), angiotensin-converting enzyme 1 (ACE1), angiotensin-converting enzyme 2 (ACE2), the (pro)renin receptor ((P)RR) or angiotensinogen (AGT) (Supplementary Figure S3), implying that renin upregulation was the main driver of the increased RAS activity, at least in the kidney.

**FIGURE 3:**
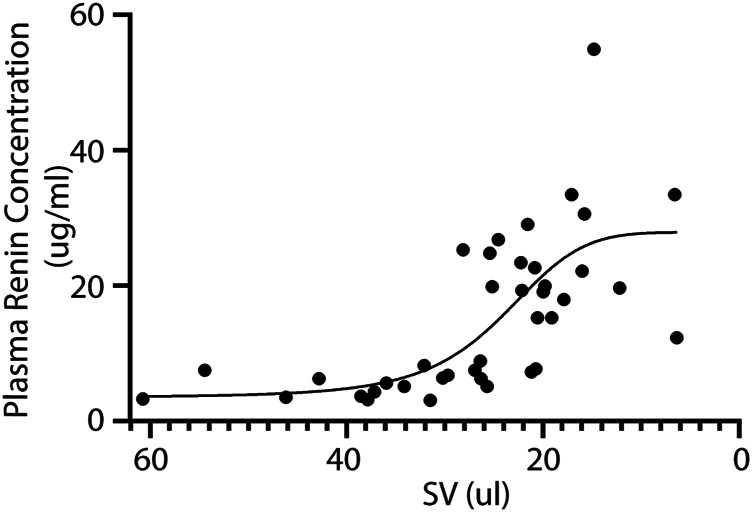
Plasma renin concentration increases upon reduced cardiac function. In line, the plasma renin levels are significantly correlated to a lower stroke volume after TAC (R2 = 0.45 and *p* = < 0.0001). SV; Stroke volume.

Next, we investigated the relation between renin expression and cardiac function in order to associate the presence of renal hypoperfusion with decreasing stroke volume. A significant positive association is present for the plasma renin concentration with decreasing stroke volume (*p* = 0.0009) (Figure 3). Although both sexes were included in our study, we did not observe any differences in renin expression between males and females (F (1,66) = 3.484, *p* = 0.07).

### Kidney Function and Structure

**FIGURE 4:**
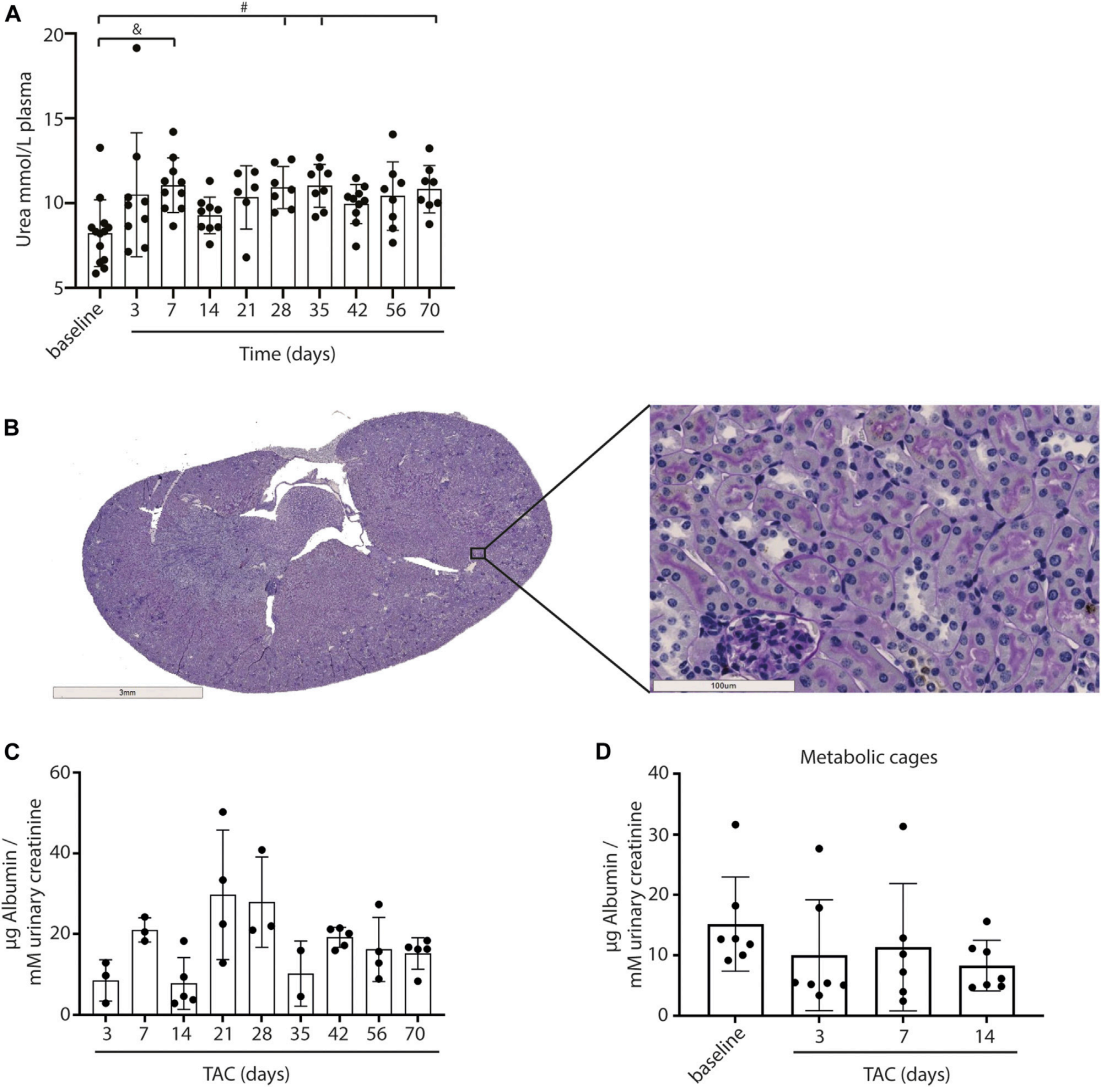
Kidney morphology, signs of damage and albumin levels. Urea levels in plasma were significantly upregulated over time after TAC versus baseline **(A)**. No structural abnormalities were observed in glomeruli and tubuli (PAS staining) **(B)**. No increase in urinary albumin (µg corrected for mM urinary creatinine) levels could be observed after TAC **(C)**. Urine albumin levels (µg corrected for mM urinary creatinine) post-TAC were not different from baseline (metabolic cages) **(D)**. Mean ± SD. #*p* < 0.05; & *p* < 0.01.

**FIGURE 5:**
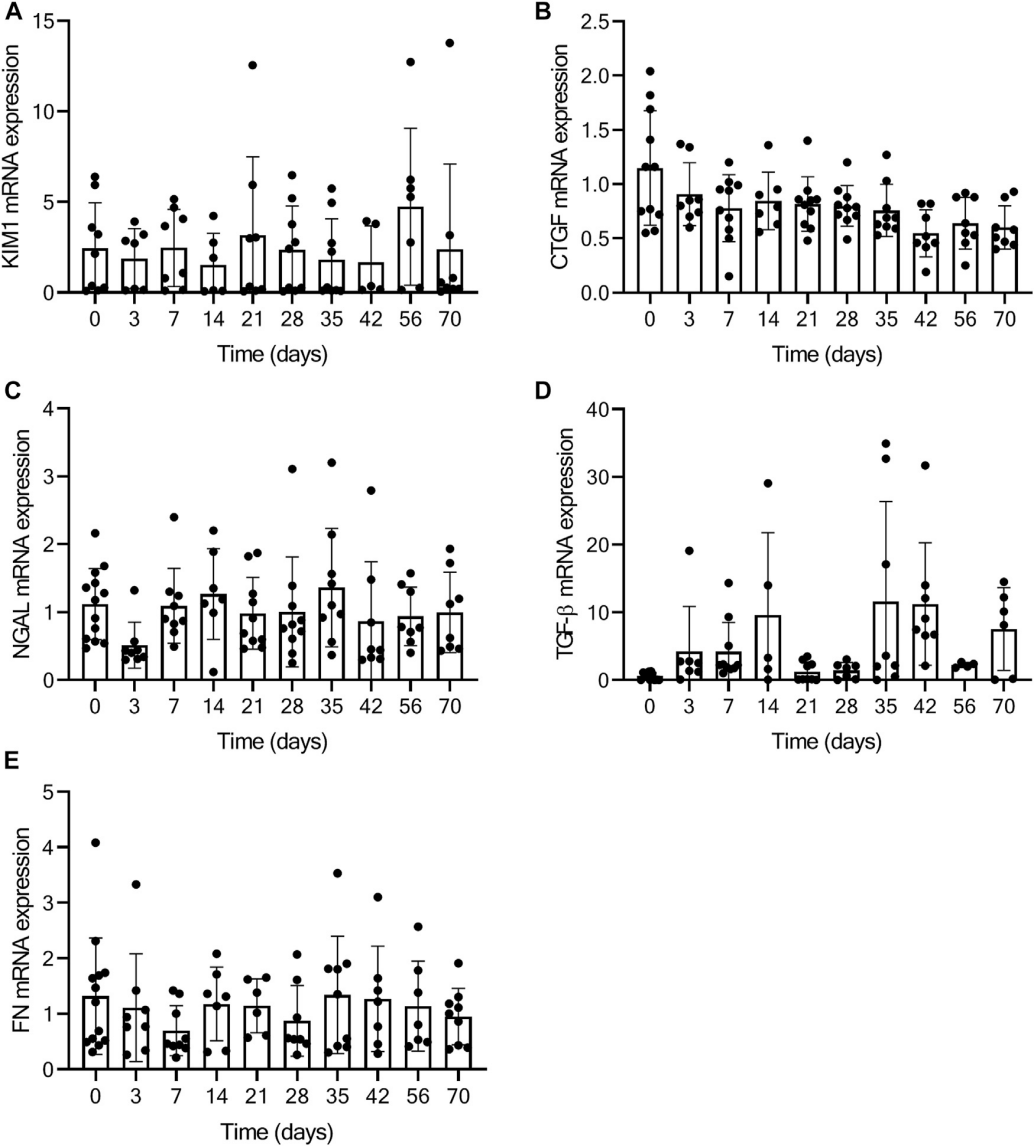
No kidney injury present based on qPCR analysis. Figure displays the relative expression of KIM-1 **(A)**, CTGF **(B)**, NGAL **(C)**, TGF-β **(D)**, and FN **(E)** at baseline (0) and over time post-TAC. No significant increase or decrease in mRNA expression is present, comparing post-TAC time points to the baseline animals.

As a measure for kidney function, we assessed glomerular function, morphological changes and albuminuria in mice exposed to TAC. From day 7 onwards mildly increased urea levels were observed, without further increase over time after TAC compared to baseline (day 7, *p* < 0.01; day 28, 35, and 70, *p* < 0.05) (Figure 4A). The increase in urea suggests a mildly reduced renal glomerular filtration as a possible consequence of heart failure development. Analyzing the PAS staining, no fibrosis, sclerosis or an indication of infiltrating immune cells was observed in either the glomerular or tubulo-interstitial compartments upon TAC at any timepoint (Figure 4B and Supplementary Figure S4). To elaborate, qPCR analysis of fibronectin (FN), connective tissue growth factor (CTGF), neutrophil gelatinase-associated lipocalin (NGAL), transforming growth factor-β (TGF-β), kidney injury molecule-1 (KIM-1) confirmed our histology data and showed no significant indications of kidney injury over time Figure 5. Incidentally, we did observe some random protein casts in the kidney medulla with no apparent relation to duration of TAC (*n* = 28/107, ranging from day 0 till day 70). No differences in albuminuria could be observed between early and late timepoints after TAC (Figure 4C). Because we were unable to obtain enough urine from all mice and were not in possession of baseline samples, we included 10 additional TAC mice to collect urine using metabolic cages during a 14 days follow-up. Again, no differences were observed for albumin excretion post-TAC compared to baseline (Figure 4D). Although both sexes were included in our study, we did not observe any differences in kidney function (F (1,84) = 2.217, *p* = 0.14) and structure between males and females. To summarize, despite the severe cardiac remodeling after prolonged exposure to TAC, there is no indication of structural damage or kidney dysfunction after prolonged exposure to TAC.

## Discussion

The influence of the cardiorenal axis in the TAC heart failure model (Tan et al., 2018) is debated. We evaluated renal function and structure in relation to renin expression in the TAC model over time. We are the first to provide an elaborate TAC study of progressive heart failure that assesses both, acute and progressive effects of heart failure on renal function in relation to renin expression in the absence of systemic hypertension. Our results indicate that in the TAC model, increased renin levels are significantly associated with reduced stroke volume. Despite very severe heart failure upon prolonged TAC we did not detect any significant functional or structural abnormalities in the kidneys. Taken together, our results indicate that in the murine TAC model, the kidneys remain intact independent of worsening cardiac function, possibly as a consequence of renin activation. Stimulation of the RAS and aldosterone by a direct reduction in renal perfusion will increase effective circulating volume and therefore to some extent maintain renal perfusion and filtration, offsetting the decrease in SV (and CO) chronically induced by TAC.

It has previously been shown that a strongly reduced stroke volume can activate RAS, leading to increased inflammation and severe renal fibrosis (Rudomanova and Blaxall, 2017; Zhao et al., 2019). For example Li et al, reported RAS activation due to cardiac pressure overload 18 weeks post-TAC (Li et al., 2018), where Zhao already reported increased albuminuria and structural abnormalities in the kidneys after 8 weeks of TAC (Zhao et al., 2019). The lack of appropriate numbers for statistical analysis of the urinary albumin measurements is a limitation of our study and might explain why we were unable to reproduce these results. However, slightly contradictory results have also been described. For instance Tan et al., show that after 10 weeks of TAC mild renal pathology is present, represented by modest albuminuria with mainly healthy glomeruli present. This mild renal pathology did not associate with the progressive cardiac remodeling post-TAC (Tan et al., 2018). This is in line with others who showed no signs of kidney fibrosis 4 weeks post TAC in a model with mild, moderate and severe aortic constriction (Richards et al., 2019). None of these studies included sequential time series to evaluate the start and progression of renal dysfunction in TAC mice. Here, in a longitudinal study, we show that kidney function and structure remain normal up till 10 weeks post-TAC. Nevertheless, our model might be too mild to already induce renal dysfunction after 70 days, therefore to observe this we might have to considerably extend our follow-up beyond 10 weeks. But with longer follow-up we risk loss of animals as mice already suffer from severe cardiac dysfunction.

Although we observe preserved renal function after TAC, we cannot ignore the consequences that high renin levels may have on the heart and kidney *via* RAS activation (Li et al., 2018). For example, angiotensin II (Ang II) is known to induce renal and cardiac fibrosis. Direct measurement of angiotensin II in tissue is very challenging and requires direct homogenization of fresh tissue (Verhagen et al., 1999), which was not feasible in the present study. Nevertheless, as renin plays an important role in the Ang II production, increased renin levels can directly stimulate cardiac fibrosis formation, and renin is reported to affect renal perfusion (Passier et al., 1996; Schroten and Gaillard, 2012; Chinnakkannu et al., 2018). Therapeutic intervention of RAS can interrupt this vicious circle. Indeed, treatment with angiotensin-converting enzyme inhibitors (ACEi) and angiotensin II receptor blockers (ARB) are proven to be protective for heart failure and prevent left ventricular stiffness in humans, most likely as a direct consequence of reduced angiotensin II levels (Wright et al., 2008). In addition ACE inhibition results in an improved cardiac and renal function in mice after TAC (Wang et al., 2016; Chinnakkannu et al., 2018).

It can be debated that the resistance of C57BL/6 mice to develop hypertension and kidney failure including proteinuria and glomerulosclerosis (Rabe and Schaefer, 2016) might have influenced our findings. For further research into the cardiorenal axis, 129/Sv mice that are susceptible for kidney failure may provide more insight; however, these mice show a very severe cardiac phenotype and are more prone to cardiac rupture after MI (Pei et al., 2017). Besides strain, high estrogen levels in female mice might help protect against kidney injury and vascular dysfunction (Dubey and Jackson, 2001; Pei et al., 2017). Estrogens have various established protective effects against oxidants, uremic toxins, microvascular dysfunction and several other pathogenic cellular and biochemical pathways (Dubey and Jackson, 2001; Pei et al., 2017). Although we didn’t observe differences between male and female mice, the inclusion of ovariectomized mice in a future study may provide answers regarding the influence of estrogens on the renal and cardiac function. To conclude, a mouse model mimicking clinical cardiorenal syndrome in patients remains challenging, especially if intrinsic characteristics like strain and sex might influence the outcome.

When using the TAC model to study heart failure it is important to take the cardiorenal axis into account, patients with reduced cardiac function often also experience CKD (Harada et al., 2018; Kumar and Seth, 2019; Patel et al., 2019; Zaleska-Kociecka et al., 2019), as it has been suggested that a decline in kidney function directly contributes to further deterioration of cardiac function in patients (Hewitson et al., 2015; Gnanaraj and Radhakrishnan, 2016). Several papers have indicated that a good animal model that properly represents the clinical manifests of CRS is lacking (Szymanski et al., 2012; Tan et al., 2018; Liu, 2019). Attempts have been made to developed such CRS models; for instance showing adverse cardiac remodeling in a severe CKD model thereby mimicking aspects of CRS4 in patients (Verhulst et al., 2017). Others have shown an experimental model of dual insults that combine myocardial infarction or doxorubicin induced dilated cardiomyopathy, followed by 5/6 subtotal nephrectomy, thereby mimicking patients with preexisting chronic heart failure and asymptomatic renal dysfunction (Liu et al., 2013; Chua et al., 2016). One could also argue that the TAC model represents primary renal malfunction (decreased renal perfusion due to reduced SV, leading to renin secretion and angiotensin formation), followed by gradual development of angiotensin-II dependent chronic heart failure, therefore modelling CRS. However further studies are needed to gain insights into the variety of bidirectional pathway interactions between heart and kidney, like hemodynamic, humoral, metabolic and cell mediated communication in animal models to overcome the current limitations of the existing models (Szymanski et al., 2012; Hewitson et al., 2015).

In conclusion, we show that progressive heart failure within our TAC model only mildly affects renal function without inducing significant structural or functional abnormalities. Our results do not support the hypothesis that heart failure due to acute cardiac pressure overload leads to rapid progressive deterioration of kidney function. Indeed, our data suggest that the TAC model is suitable to test the function of novel proteins in or therapeutics for heart failure without an influence of kidney dysfunction other than renin release.

## Data Availability

The raw data supporting the conclusion of this article will be made available by the authors, without undue reservation, to any qualified researcher.
