# Supplementary material for: Mildly Increased Renin Expression in the Absence of Kidney Injury in the Murine Transverse Aortic Constriction Model
Source: Front Pharmacol. 2021 Jun 15;12:614656. doi: 10.3389/fphar.2021.614656 (PMC8239225; doi:10.3389/fphar.2021.614656)
Supplement: Supplementary file 1 [file DataSheet1.pdf]

A

	Male	Female
**Baseline (no surgery)**	6	7
**TAC surgery**	50	54
**Excluded based on flow ratios**	3	3
**Excluded based on inconsequent echo data**	2	2
**Died during follow up after TAC surgery**	7	5

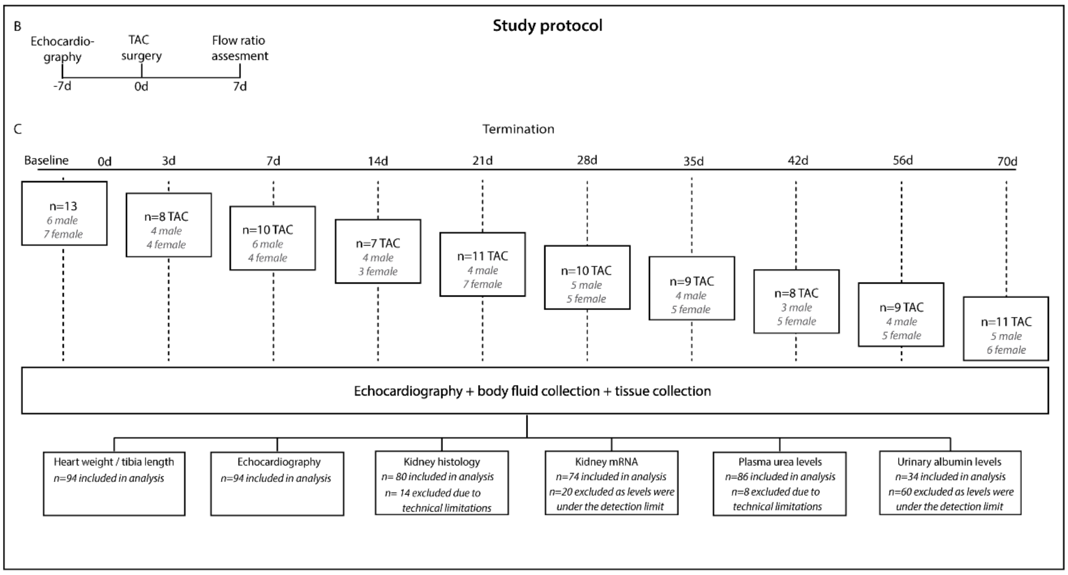

Fig. S1. Study protocol TAC mice. A) number of mice included as control (no surgery) and TAC surgery. In addition, the number of mice that were excluded based on the carotid Doppler flow measurements, no TAC present and the number of mice that are lost during follow up as direct consequence of surgery (extreme inflammation in surgical area, ruptured aorta, sudden cardiac arrest within 0-7 days post-TAC). No mice were lost during later stages of the experiments. B) Start of the experiments. **c**) termination protocol.

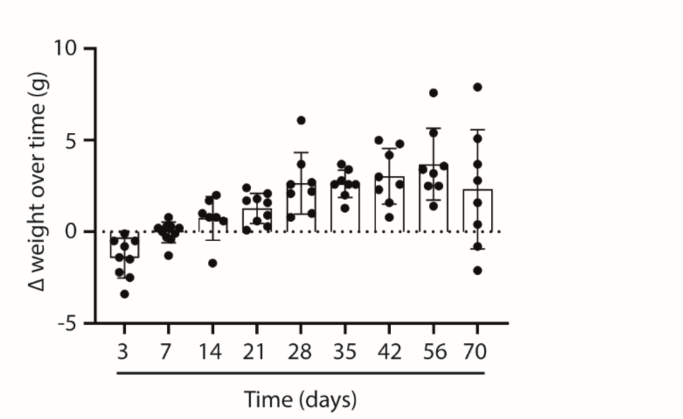

Fig. S2. Change in weight over time post-TAC. Figure displays the change in body weight compared to the baseline weight over time post-TAC. A decrease in body weight is present at day 3 post-TAC as consequence of surgery, at day 7 mice regained their baseline weight. After 7 days the mice show a weight gain until the end of the experiment at 70 days.

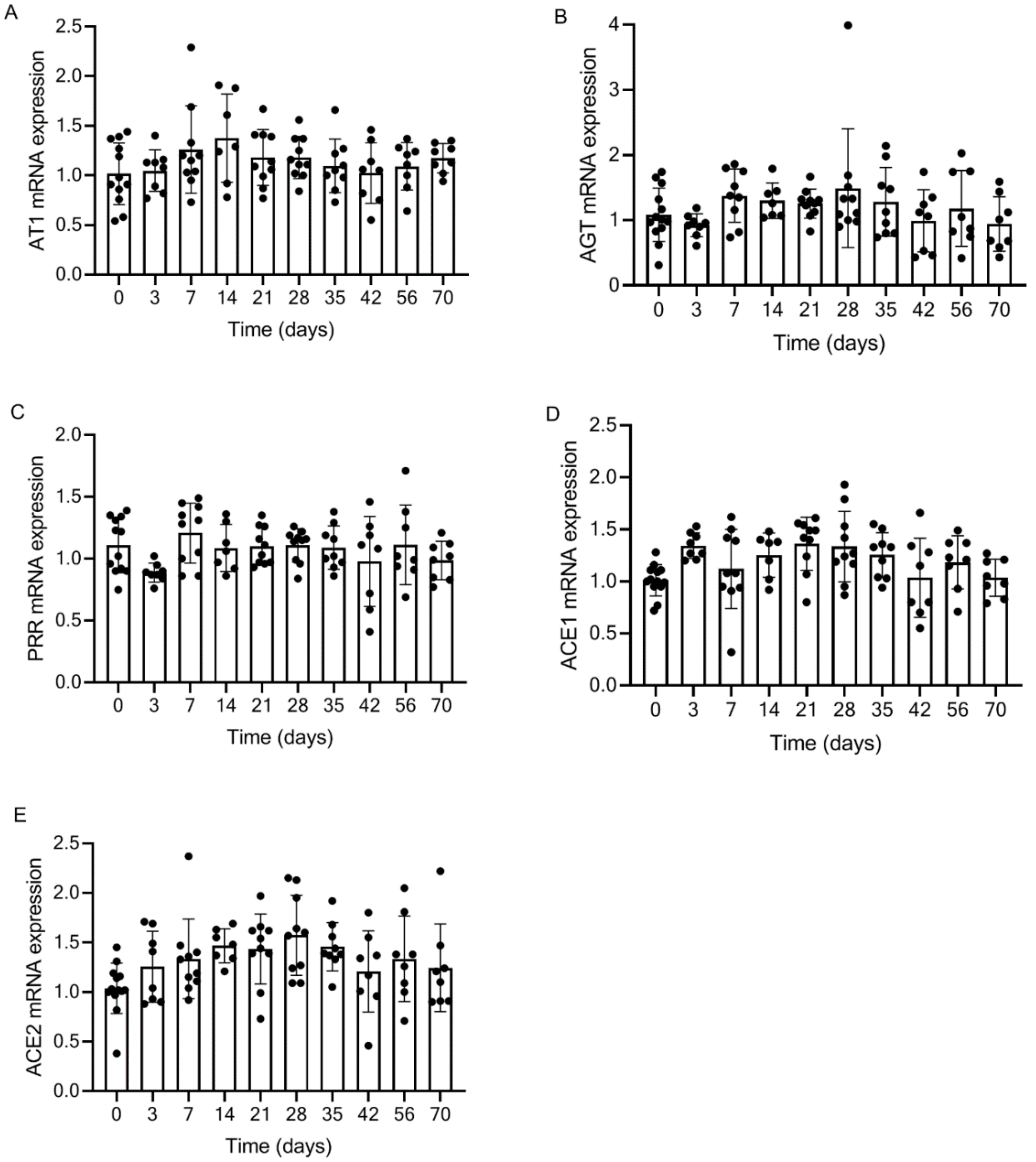

Fig. S3. No increase in renal mRNA levels of RAS components. qPCR analysis of RAS components in renal tissue showed no differences over time post-TAC for angiotensin 2 receptor type 1 (AT1) (A), angiotensinogen (AGT) (B), (pro)renin receptor ((P)RR) (C), angiotensin-converting enzyme 1 (ACE1) (D) and angiotensin converting enzyme 2 (ACE2) (E). Mean±SD.

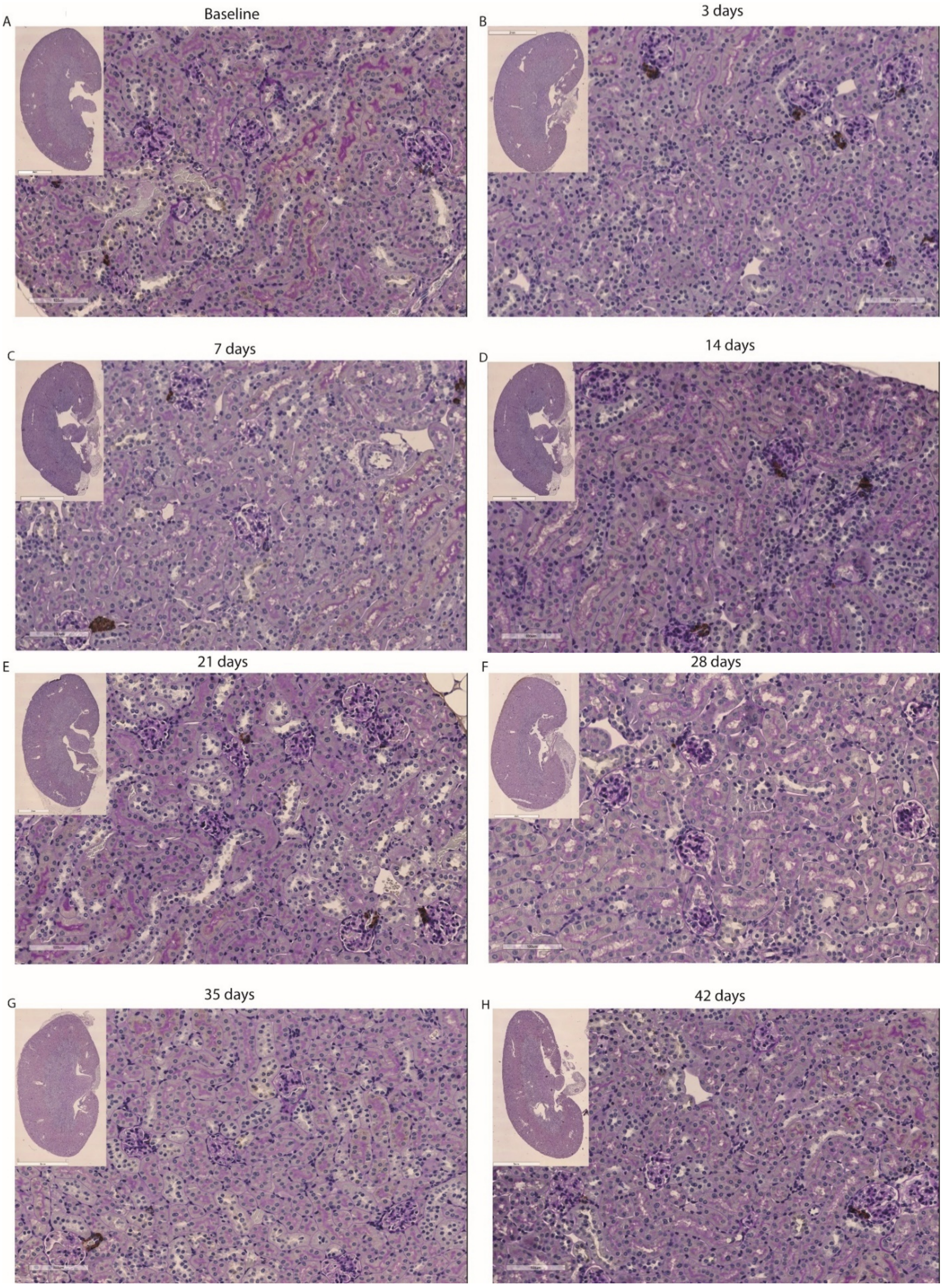
A
Baseline
B
3 days
C
7 days
D
14 days
E
21 days
F
28 days
G
35 days
H
42 days

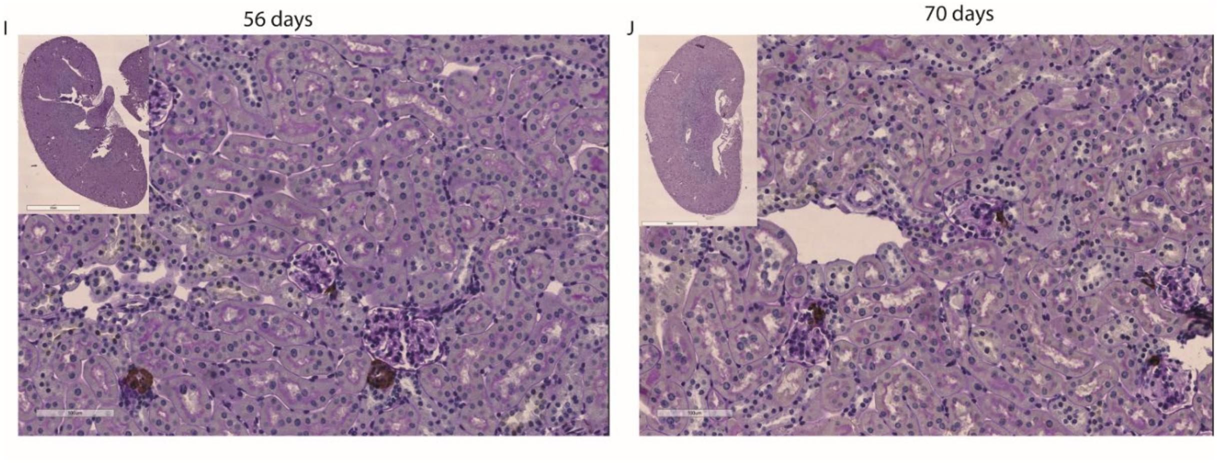

Fig S4. Representative pictures of the Renin (brown, indicated with arrows) and PAS staining (purple) over time. No structural abnormalities were observed in glomeruli and tubuli (PAS staining) at any time point post-TAC.

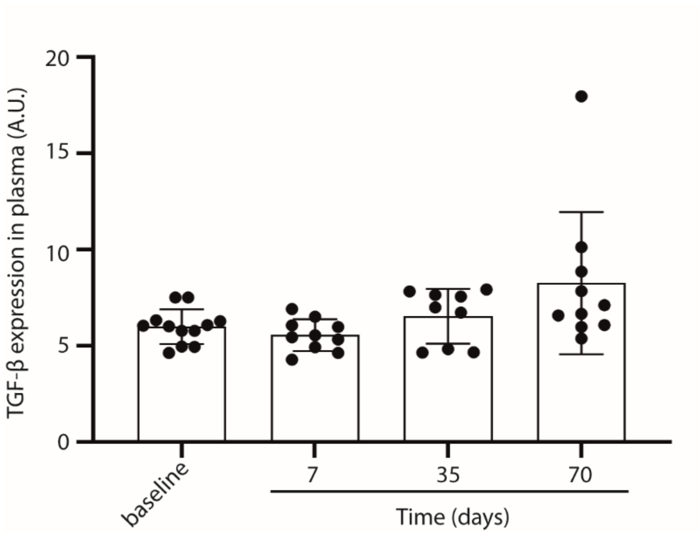

Fig S5. Circulating levels of TGF-β. With a Proximity Extension Assay (PEA) technology we measured the TGF-β levels. No differences could be observed between the baseline and post-TAC animals.
